# Smartphone-Based pH Sensor for Home Monitoring of Pulmonary Exacerbations in Cystic Fibrosis

**DOI:** 10.3390/s17061245

**Published:** 2017-05-30

**Authors:** Alexander Sun, Tom Phelps, Chengyang Yao, A. G. Venkatesh, Douglas Conrad, Drew A. Hall

**Affiliations:** 1 Department of Electrical and Computer Engineering, University of California, San Diego, La Jolla, CA 92093, USA; acs009@ucsd.edu (A.S.); tphelps4432@gmail.com (T.P.); yaobryce@gmail.com (C.Y.); agvenkatesh@hotmail.com (A.G.V.); 2 Department of Medicine, University of California, San Diego, La Jolla, CA 92093, USA; dconrad@ucsd.edu

**Keywords:** biosensor, Cystic Fibrosis, pH, point-of-care, smartphone, Iridium Oxide

## Abstract

Currently, Cystic Fibrosis (CF) patients lack the ability to track their lung health at home, relying instead on doctor checkups leading to delayed treatment and lung damage. By leveraging the ubiquity of the smartphone to lower costs and increase portability, a smartphone-based peripheral pH measurement device was designed to attach directly to the headphone port to harvest power and communicate with a smartphone application. This platform was tested using prepared pH buffers and sputum samples from CF patients. The system matches within ~0.03 pH of a benchtop pH meter while fully powering itself and communicating with a Samsung Galaxy S3 smartphone paired with either a glass or Iridium Oxide (IrOx) electrode. The IrOx electrodes were found to have 25% higher sensitivity than the glass probes at the expense of larger drift and matrix sensitivity that can be addressed with proper calibration. The smartphone-based platform has been demonstrated as a portable replacement for laboratory pH meters, and supports both highly robust glass probes and the sensitive and miniature IrOx electrodes with calibration. This tool can enable more frequent pH sputum tracking for CF patients to help detect the onset of pulmonary exacerbation to provide timely and appropriate treatment before serious damage occurs.

## 1. Introduction

Cystic Fibrosis (CF) is an inherited chronic disease that adversely affects the lungs and digestive systems of about 30,000 children and adults in the United States, as well as approximately 70,000 people worldwide [1]. Due to the abnormally viscous secretions in their lungs and pancreas that lead to inflammation and organ damage, CF patients often suffer from chronic lung disease, lung infection, bronchiectasis, and malnutrition [2]. While there is currently no cure for CF, fortunately, advances in both screening and treatment have drastically increased the average lifespan and improved their quality of life. However, those with CF are still constantly at risk of pulmonary exacerbations, which can cause irreversible, life shortening destruction of the airways, with obstructive lung disease being the primary cause of morbidity and mortality [1,3]. Hence, people living with this lifelong disease must follow a regular treatment routine to stay healthy and maintain optimal lung function, requiring frequent physician visits to test and clear airway infections.

Unfortunately, current tools, such as clinical culturing, take days to obtain results, and are not able to detect whether a patient is undergoing an exacerbation, leaving physicians to make choices about antibiotic treatment based on trial and error. Recent studies have shown that a decrease in pH of exhaled breath condensate, as well as increase in fermentation products and upregulation of protein biomarkers in sputum, is associated with and precede exacerbation events [4,5,6,7,8,9]. Therefore, monitoring pH levels as well as other biomarkers over time would allow patients and physicians to make informed decisions about how to treat airway infections before exacerbations progress and cause further damage.

**Figure 1:**
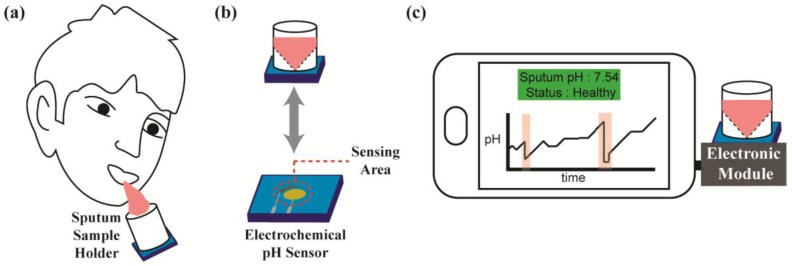
Overview of the smartphone-based pH sensor platform with (**a**) sputum collection via a sample holder, the bottom of which is (**b**) a reusable electrochemical pH sensor. (**c**) The entire holder attaches to an external electronic module that plugs directly into the headphone port of the smartphone to make and track measurements via a custom app.

While very few CF patients have the flexibility and means to participate in frequent advanced diagnostic tests for airway infections typically only available in ERs or doctors’ offices, almost all have access to smartphones. Hence, in this paper, we present one of the first low-cost, point-of-care (POC) smartphone-based medical tools for at-home monitoring of CF exacerbations that provides physicians with long-term actionable data to adjust therapy. The entire platform (Figure 1) consists of a smartphone, an electronic module that conducts electrochemical pH measurements, and a sample holder mounted on top of a modified screen-printed electrode. The module plugs into the audio jack of a smartphone, making it hardware compatible with all makes and models and able to extract power directly from the phone obviating the need for a battery or external power source. This device allows CF patients to track their condition daily from home, thus greatly increasing the specificity with which exacerbations can be tracked and treated. Furthermore, their critical information can be collected and sent to medical professionals who can then adjust the treatment regime for infections. The smartphone also incorporates validated quality of life questionnaires, such as the UCSD Shortness of Breath Questionnaire (SOBQ) and the CF Questionnaire Revised (CFQR) to correlate disease activity with the quantitative data collected [10,11,12,13]. This peripheral is also intended to be a platform device that, with slight modification, can easily incorporate further electrochemical sensors for other CF biomarkers, the assays for which are in the progress of being developed. 

## 2. Related Work

While there have been many sensitive and state-of-the-art POC-oriented biosensors for Cystic Fibrosis, these devices tend to focus solely on the diagnosis of the disease, rather than monitoring the health and detecting the presence of infection in those already diagnosed [14,15,16,17,18]. Since the platform presented here is unique in that it is meant to be a much more frequently used tool to track a CF patient’s daily pH levels, rather than a one-time use test, the portability of the device is crucial to its operation and overall practicality. While other POC devices meant for diagnosis via immunoassays use microliter volumes of invasive samples such as drops of blood or serum, which often require advanced microfluidics to handle and process [18,19], the specific application targeted in this work allows for the use of sputum, which can be non-invasively extracted in larger volumes (>1 mL). Hence, a much simpler collection scheme can be employed to handle the biological sample. Furthermore, an electrochemical method of detection is used since the hardware required is comparatively more scalable than other methods. For example, there are several smartphone-based pH meters that use optical measurement techniques exploiting the internal camera and flash of the smartphone [20,21,22,23,24,25]. These systems are typically prone to interference from environmental lighting or depend on the precision of colorimetric test strips, which are often only intended to give a rough estimate of pH. Achieving high sensitivity requires external lenses, filters, and light sources—all of which are difficult to miniaturize in terms of size and power when compared to electrochemical sensing methods. However, one of the major challenges in electrochemical pH measurements is the selection of the electrode type, where small size, fabrication difficulty, sensitivity, and stability are traded off with each other [26,27].

While large glass fritted electrodes are still common in laboratory use, there are currently multiple options for state-of-the-art electrochemical pH sensors more suitable for point-of-care applications such as metal oxide film, conducting polymer, and ion selective field effect transistor (ISFET) electrodes [28,29,30,31]. Metal oxides including tungsten, platinum, lead, iridium, ruthenium, and antimony oxides are extremely robust and can withstand harsh environments, high temperature, and high pressure. Specifically, Iridium Oxide (IrOx) has been demonstrated to have a higher sensitivity and faster response time than that of glass [32,33,34]. Conducting polymers such as polyaniline exhibit less instability and drift overtime than metal oxide electrodes and, hence, require less frequent calibration for comparable accuracy [30,35]. ISFETs can dramatically reduce the size and area of sensors, but, due to their fabrication cost and complexity, they are more appropriate for applications requiring nanoscale or arrayed sensors rather than for instances where macroelectrodes would suffice [28,31].

On the electronics side, ever since the audio-jack was first demonstrated as a viable communication and power transfer port on the smartphone [36], many health-related sensors have been similarly integrated with the smartphone to enable novel mobile health tools [27,37,38,39]. However, the intrinsically low and inconsistent maximum output power of different headphone ports limits the types of sensors that can leverage the harvesting technique to those with low enough power consumption. Furthermore, most harvesting designs, including commercial ICs (e.g., NXP OM13069), settle for suboptimal power transfer efficiency across different makes and models of smartphones, further lowering the power available to the sensing electronics and reducing the device’s overall compatibility with different host devices [40]. Hence, many of these sensors that require extensive power often use an external battery minimizing the advantage of using the headphone port in the first place. This work, instead, is based on previous augmented power harvesting designs that address this issue through implementing automatic power transfer efficiency optimization circuits and algorithms. In the following sections, our design of a more efficient power harvesting electrochemical platform coupled with an inexpensive and reliable Iridium Oxide (IrOx) electrode is discussed and addresses many of these issues.

## 3. Design of a Smartphone-Based Sensor Platform

The smartphone-based electrochemical sensing platform discussed below is based on a potentiostat developed previously with modifications made to tailor the device for POC pH sensing [41]. The platform consists of three main items: (1) the peripheral electronic module, (2) a point-of-care oriented pH sensor, and (3) the smartphone application, which communicates with and manages the data collected from the sensor.

### 3.1. Smartphone Peripheral pH Sensor Module

**Figure 2:**
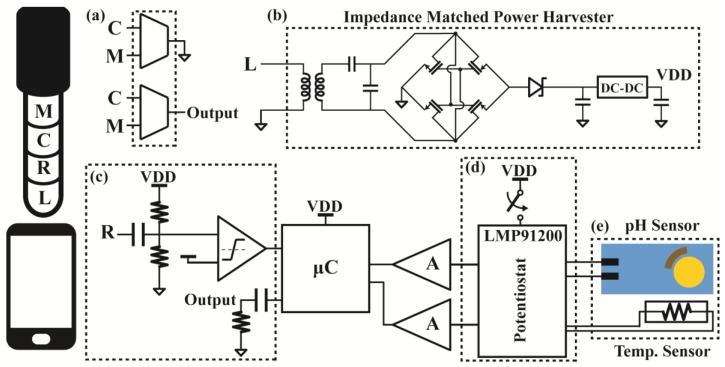
Block diagram of the electronic pH module that communicates with the smartphone and interfaces with the sensors. The module contains: (**a**) multiplexers to select the correct pins to connect to the common and microphone ports, (**b**) an impedance matched power harvester that rectifies and transforms a signal from the left audio output to power the entire module, (**c**) bidirectional AC coupled communication with the smartphone that sends back the gathered data, a (**d**) readout and signal conditioning circuitry, and (**e**) pH and temperature sensors.

The peripheral module (Figure 2) plugs directly into the smartphone’s headphone jack to harvest power and facilitate bidirectional communication between the phone and the module. By using the headphone port instead of other interfaces available on the smartphone (e.g., USB or Lightning port), the module can work across all types of smartphones, even tablets and some feature phones, regardless of the make or model. Furthermore, since power can be efficiently harvested from the headphone jack, the user does not have to deal with the hassle of charging, managing, or replacing additional batteries common in devices that use Bluetooth. Even though the AC coupled nature of the audio port requires additional circuitry in order to utilize the phone’s energy, this harvesting method has been shown to provide enough power for similar low-power sensors with small form factors.

This module also contains an analog front-end that handles measuring data from the sensors. While the actual mechanism of electrochemical pH sensing varies depending on the type of electrode used, such as standard glass electrodes, ion selective field-effect transistors (ISFET), or oxide-film electrodes, the measurement method is still the same in all cases. Due to the presence of hydrogen ions inside a test solution, a potential difference builds up between two electrodes submerged in the solution that have been modified to specifically detect hydrogen. By measuring this voltage without disturbing the electrochemical cell, the pH level of the solution can be determined. Finally, an on-board microcontroller relays the information back to the smartphone for analysis. Due to the AC coupling, a frequency modulation technique is used to transmit and receive data.

#### 3.1.1. Efficient Power Harvesting

The smartphone’s audio port consists of four connections: left (L) and right (R) audio outputs, microphone (M) input, and common (C). The AC coupling restricts the module from directly tapping into the DC power of the audio output. Hence, to provide power, the smartphone outputs a sinusoidal tone at a single adjustable frequency (5–20 kHz) on the left channel. The module boosts this low voltage (~200 mV) tone with a transformer, passes it through a voltage rectifier, and then steps it up to 3.3 V with a DC-DC converter to power the rest of the board.

Different smartphones, however, have different audio driver characteristics meaning that the output impedance and power available from the headphone port can vary. This inconsistency can cause poor impedance matching between the different phones and the module leading to inefficient power transfer and inadequate power delivered to the module. To account for this variation, an impedance matching network is introduced into the power harvesting circuitry that can be tuned by adjusting the frequency of the tone provided by the smartphone. Hence, regardless of the smartphone used, the module can adjust its own impedance to match the output impedance of the smartphone, thereby always providing optimum power transfer to the module, improving the efficiency from ~50% up to 85% of the available power [40]. This architecture has also been shown to harvest 3× more power than a commercial off-the-shelf chip using the same phone [42].

#### 3.1.2. pH and Temperature Measurements

A low-power, small form-factor, commercial, analog front-end (LMP91200, Texas Instruments, TX, USA), which can interface with a variety of pH electrodes, was chosen to measure the potential difference that develops between the two terminals of the electrode in the test solution. Essentially, the component contains a high input impedance buffer (~100 TΩ) that can probe the voltage of the electrochemical cell, which has a large impedance (~100 MΩ). The output of the buffer is amplified via a non-inverting amplifier (OPA2333, Texas Instruments, TX, USA) with a gain of 5 and read by the 8-bit analog to digital converter (ADC) inside the microcontroller (PIC16F690, Microchip, Chandler, AZ, USA).

It is desirable for the module to be able to use different types of pH electrodes, each with varying voltage ranges, to compare multiple electrodes. To accommodate this large variation of voltage ranges while still maximizing the accuracy of the measurement circuitry, the common-mode voltage of both the pH electrode and the amplifier can be adjusted for each different type of electrode. Furthermore, since the pH measurements are highly dependent on the temperature, a thermistor biased by a current source on the LMP91200 is dipped into the solution under test to measure its temperature, which is used to compensate the pH data for any thermal shifts that occur. By switching from the onboard thermistor to an external sensor via a 2.5 mm audio port, the module is also compatible with pH electrodes integrated with internal temperature sensors. The smartphone can then easily use the temperature readings and the Nernst equation to calibrate the pH.

#### 3.1.3. Communication via Frequency Modulation

Another discrepancy that exists between some smartphone models is that the microphone and common terminals are sometimes swapped in certain phones, such as the Motorola Moto X. To account for this variation, multiplexers are placed on both the microphone and common channel to be able to interchange them if necessary. Since the microphone pin will be at a higher voltage than the common pin due to the presence of the current source at the microphone output, a comparator tied to both allows the module to determine the correct orientation automatically. Once the microphone channel has been properly set, communication can take place between the device and its host.

**Figure 3:**
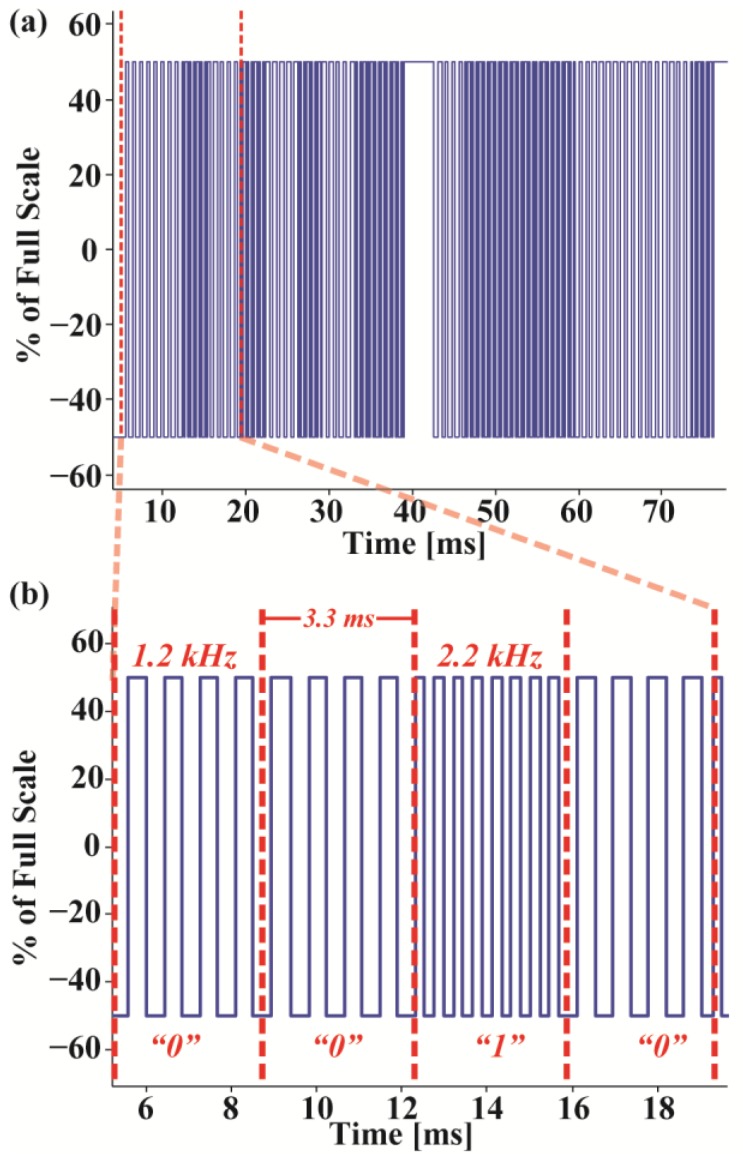
(**a**) A packet with frequency shift keying modulation and (**b**) a portion of the data decoded back to its original bits with zeros at 1.2 kHz and ones at 2.2 kHz.

However, the AC coupling of both the microphone and audio channels blocks low frequency signals from being transmitted between the smartphone and the module. While simply sending packets of data via bit banging or a UART has been demonstrated to work in short bursts, these methods typically suffer from reliability issues. Distortion of these data packets originates from the signal dependent shaping of the waveform that occurs on the audio channels causing drift and amplitude variation. To avoid this effect, packets are instead constantly modulated using frequency shift keying (FSK) as shown in Figure 3, where the frequency component of the signal holds the data. Hence, this modulation scheme remains robust even in the presence of shifts in the DC voltage or attenuation of the signal amplitude. To minimize the power requirement of the microcontroller, low 1.2 kHz and 2.2 kHz frequency signals are used to represent “0” and “1”, respectively. This implementation achieves a bit rate of 300 bits per second, which is higher than is necessary due to the low data rate nature of the sensors.

### 3.2. Point-of-Care Oriented pH Sensor

Since several types of the potentiometric pH sensors discussed earlier would each be useful under different scenarios and conditions, instead of designing the platform around a specific type of sensor, the device in this work is meant to be compatible with a wide range of potentiometric pH sensors. This strategy also allows the device to incorporate ongoing improvements in ion-selective sensors through replacement of the electrode without a full platform redesign. In this work, both standard glass electrodes and Iridium Oxide modified screen-printed electrodes are used to demonstrate the platform’s effectiveness as a POC sensor and its compatibility with multiple types of pH sensors. The glass pH probe was chosen since, due to its excellent sensitivity, selectivity, stability, and long lifetime, it is the gold standard used in laboratory settings to determine the pH of an aqueous solution [43]. However, the semi-permeable glass membrane that gives the probe these properties is very fragile, expensive to fabricate, and difficult to miniaturize for POC applications. Iridium oxide was selected for the POC oriented sensor because it is a well-established and thoroughly studied sensor type allowing us to obtain a highly sensitive and stable electrode with simple and inexpensive fabrication steps. IrOx electrodes built on sputtered metal or wire (gold or platinum) have been shown to have high sensitivity (63.5 mV/pH), 0.02 pH accuracy, relatively long lifetime (~1 month to years), and low drift (0.1 mV/day) [32,33,43]. Furthermore, IrOx film is often used to coat invasive neural probes implanted under the skin or directly in the brain, with extensive research demonstrating that IrOx is highly biocompatible [44,45,46,47]. Hence, IrOx is a safe and ideal electrode material for at-home pH monitoring, especially since the electrode surface in this application does not come into direct contact with the user. Additionally, to make these electrodes easier and less expensive to fabricate, commercial gold screen-printed electrodes (SPE) were used as the base metal before modifying with IrOx. SPEs are self-contained as they have both the working and reference electrode on the same substrate and are already mass produced.

## 4. Materials and Methods 

**Figure 4:**
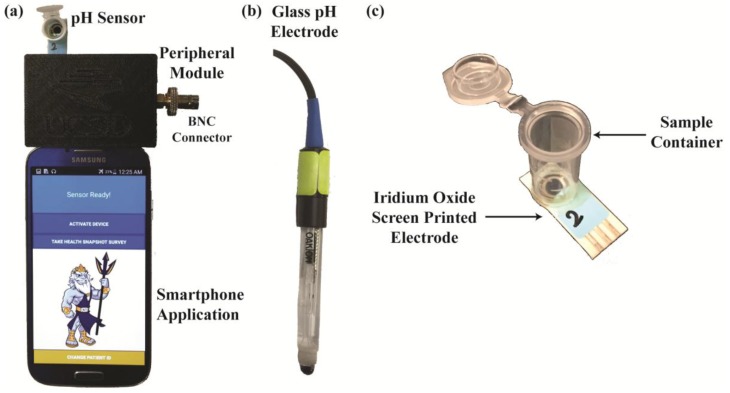
(**a**) Entire platform with the peripheral module inside a 3D-printed case, pH sensor chip, and smartphone running the custom CF application. Both (**b**) standard glass pH electrodes and (**c**) Iridium Oxide modified screen-printed electrodes can be used with this system.

A 3.8 cm × 5.8 cm printed circuit board (PCB) of the electronic module described previously was constructed. Assembly consists of soldering the components onto a custom printed circuit board, programming the on-board microcontroller, and mounting the electronics in a case. The device has standard BNC, 2.5-mm, and 3.5-mm connectors to attach both glass pH electrodes and the screen-printed electrodes as well as their corresponding temperature sensors (Figure 4). For all calibration and comparison testing, both standard pH solutions (Orion 910104, 910107, 910110, Thermo Fischer Scientific, Waltham, MA, USA) and phosphate buffered solutions (PBS) at different pH levels were used. A benchtop pH meter (Orion Star A211, Thermo Fischer Scientific, Waltham, MA, USA) measured the actual pH of each solution and set the nominal level for each.

### 4.1. Preparation of Iridium Oxide Electrodes

The Iridium Oxide film was electrodeposited onto gold SPEs (250AT, DropSens, Asturias, Spain) using an established deposition solution [33]. The solution consists of 4.5 mM Iridium tetrachloride (#516996, Sigma-Aldrich, St. Louis, MO, USA) with 0.3% hydrogen peroxide (#H325, Thermo Fischer Scientific, Waltham, MA, USA) and 40 mM oxalic acid dehydrate (#75699, Sigma-Aldrich, St. Louis, MO, USA). This working solution was left to stabilize at room temperature for two days. To electrodeposit the film, a 50 µL droplet was applied to each SPE and scanned with cyclic voltammetry from −0.1 to 1 V at 20 mV/s for 25 cycles, roughly 45 min. A commercial potentiostat (750E, CH Instruments, Austin, TX, USA) was used to conduct the cyclic voltammetry deposition. After rinsing in deionized (DI) water, the electrodes were conditioned in a neutral pH tris buffer for 2 days before use. Each of these electrodes were calibrated using the standard pH buffers. Between different tests, the electrodes were rinsed with DI water, air dried, and kept in a petri dish at room temperature to simulate practical storage conditions.

### 4.2. Validation of Module and Iridium Oxide Electrode

After a single calibration using standard pH buffers, the electronic module powered and controlled by a Samsung Galaxy S3 smartphone paired with the Oakton pH Probe (WD-35811-74, Oakton Instruments, Vernon Hills, IL, USA) was used to measure the PBS solutions at different pH levels from pH 5.9 to 8.08. Repeated measurements were made daily over the course of a week without recalibration and compared against the nominal pH set by the commercial pH meter and probe. Similarly, the IrOx electrodes were used to measure two sets of buffers across 5 days. The solutions were tested with the same setup once a day for an entire week without recalibration with each electrode stored dry.

### 4.3. Patient Sputum Samples

Sputum samples were collected from three CF patients having undergone sputum induction using a standard nebulizer with hypertonic saline. Soon after the samples were gathered, each was initially measured using the smartphone-based pH platform coupled with a glass electrode to track the change in pH and temperature over time. Each sample was then measured with both the glass electrode and IrOx electrodes using the smartphone platform as well as the pH meter. None of these samples were diluted or processed before testing. For calibration of the IrOx electrodes, 200 µL aliquots of a single sputum sample were repeatedly spiked with 1 µL volumes of NaOH and HCl concentrations between 100 µM–1 mM diluted in DI water. Through this addition of the strong acid and base and by using the glass probe to set the nominal values, the pH of the sputum medium was artificially adjusted to obtain a calibration curve for the IrOx electrodes. In total, no more than 10 µL of either acid or base was added to each aliquot, thus limiting error from dilution of the sputum.

## 5. Results and Discussion

### 5.1. Performance of Electronic Module

The electronic module consumes a total maximum power of 6.2 mW during its measurement operation (~120 s) with the microcontroller and analog-front-end dominating this number. Assuming that a typical smartphone battery has a capacity of ~1500 mAh, even the electronic module running a few times a day would have a negligible effect on the phone’s battery life. The total integrated noise (0.1–10 Hz) of the analog-front-end including the amplifier is 70 µV_RMS_, which sits below the quantization levels of the ADC (~3.6 mV).

**Figure 5:**
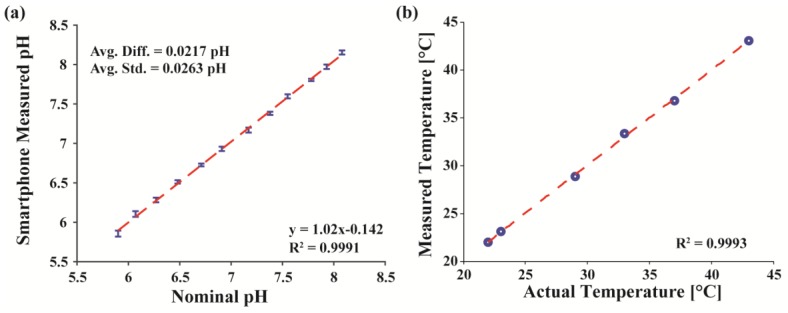
(**a**) Averaged measured pH data taken over one week (*n* = 7) for different nominal pH solutions ranging from 5.9 to 8.08 pH. The dotted line is a linear fit of the averaged data; (**b**) Temperature measured by the module compared to actual temperature recorded by a commercial thermometer from 20 to 45 °C.

Measured pH data taken by the smartphone-based sensor of all the PBS solutions across several days are averaged and plotted in Figure 5a. The average difference between the measured and the nominal pH values across all solutions is 0.022. The standard deviation of measurements of a single solution across all seven measurements is approximately 0.026 pH. Hence, when using the glass electrode, the smartphone-based pH platform matches within ~0.03 pH of the benchtop pH meter over the timespan of a week without daily recalibration. Not only does this show that the smartphone-based tool matches well with the benchtop meter, but also it suggests that, when using glass probes, frequent or full calibration is not required.

To test the onboard temperature sensing, the module’s thermistor, after a three-point calibration, was placed on top of a hot plate and measured at various other temperatures from 20 to 45 °C using the smartphone platform. This data (Figure 5b) was compared to measurements made by a standard commercial IR thermometer (Lasergrip 1080, Etekcity, Anaheim, CA, USA) with an average temperature difference of 0.15 °C. While temperature was used to calibrate the pH measured by both the pH meter and the module (Figure S2), temperature calibration was often found to be unnecessary since the environmental conditions and measurement procedures used were kept constant for many of these experiments.

### 5.2. Iridium Oxide Electrodes

**Figure 6:**
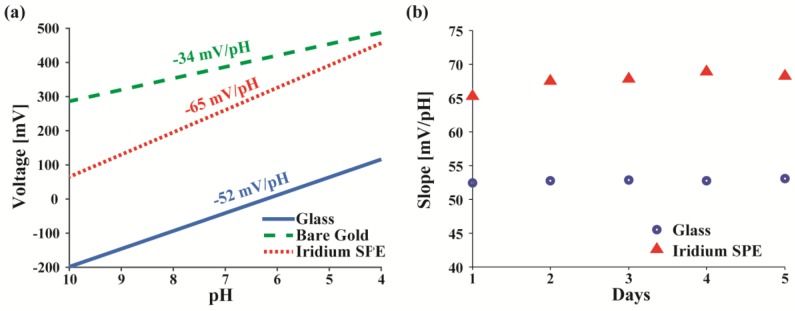
(**a**) Calibration curve of glass, bare gold SPE, and Iridium-Oxide electrode; (**b**) pH slope drift over several days for different electrode types.

The calibration curve from 4–10 pH of the IrOx electrode plotted with other electrodes in Figure 6a demonstrates the voltage ranges and sensitivity in mV per pH of each electrode. As expected, the bare gold electrode has the worst sensitivity, while the glass and IrOx perform well. The IrOx electrodes have a higher sensitivity than that of glass with a 25% steeper slope. 

One concern with IrOx film electrodes is that their absolute voltage response changes between samples containing varying amounts of salts (i.e., the sample matrix). Testing with both a set of commercial pH solutions and a set of pH-adjusted PBS solutions that contain higher concentrations of KCl and NaCl, the glass electrodes and IrOx SPEs vary on average 0.04 and 0.52 pH in the 6–8 pH range, respectively between the two sets. Hence, when moving to a different buffer, these IrOx SPEs need to be recalibrated. Once properly calibrated with standard solutions, another possible issue that can arise is the drift of electrode’s response over time after storage and repeated use. Figure 6b shows the variation of the slope (mV/pH) of the IrOx SPE compared to that of glass. The slopes of IrOx SPEs vary a maximum of 5.57% (3.64 mV/pH) over the 5-day period compared to the 1.1% (0.56 mV/pH) of the glass probe. Hence, by using a single point pre-test calibration with a standard buffer, both electrodes are relatively stable with drifts of approximately 0.004 pH/day and 0.032 pH/day for the glass probe and IrOx SPE, respectively. Further reliability improvements can be made by introducing a multi-point calibration every few days.

### 5.3. Live Sputum Test

**Figure 7:**
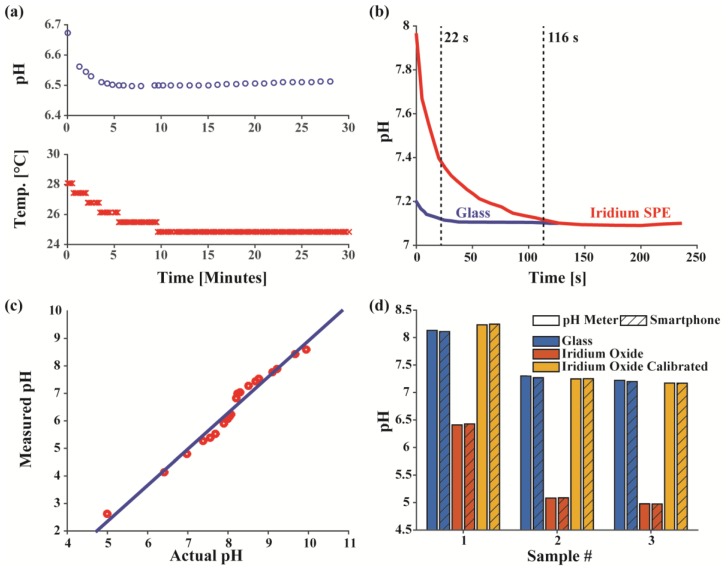
(**a**) pH and temperature settling over time of a fresh sputum sample; (**b**) Settling time of glass and Iridium Oxide electrode; (**c**) Sputum spiked to different pH levels measured with both glass and Iridium Oxide electrodes; (**d**) Actual sputum samples measured and calibrated.

The settling time plot (Figure 7a) of both the pH and temperature of a sputum sample measured immediately after collection with a glass probe and temperature sensor demonstrates that it takes at least 10 min for the sample to reach equilibrium. In the standard buffer solution tests discussed previously and seen in the pH over time plot (Figure 7b), which was recorded immediately after the buffer sample was applied, the IrOx electrodes take roughly 5× longer to settle than the glass electrode, extending measurement time from 20 s to 2 min. While the increased measurement time is not ideal for point-of-care applications, due to the temperature change of collected sputum, the settling speed is still fast enough to not affect the overall length of actual sputum testing. Being much longer than the response time of the IrOx electrodes, this settling time with the sputum sets the lower bound on how long the samples need to be incubated on the sensors surface for reliable measurements. Hence, the longer settling time of the IrOx electrodes does not affect the duration of the actual sputum assay. The intrinsic settling time of the IrOx electrodes deviates from that of others found in literature, where these electrodes often reach the final pH as quickly as or faster than standard glass electrodes. One likely explanation is that the screen-printing method used to fabricate these commercial gold electrodes results in both slower electron transfer kinetics and a vastly different oxide film formation on the electrode surface, which greatly affects its performance and characteristics.

Since sputum will contain different constituents than those of the standard buffers used for characterizing the system, to properly calibrate the IrOx electrodes, a single sputum sample was spiked to different pH levels to create a calibration curve (Figure 7c). The spiked sputum data not only allow the IrOx sputum data to be mapped back to the actual pH values, much like the initial calibration curves in standard pH buffer, but also demonstrate that the response of the IrOx electrodes to pH is still approximately linear. Hence, while the response of the IrOx electrodes in sputum do not coincide exactly with that of the laboratory buffers, the relative voltage change is approximately the same in both mediums, so, even without full calibration, the pH change of a patient’s sputum over time still offers accurate and compelling data. 

The pH measurements of each sample with all four combinations of electrodes and tools are shown in Figure 7d. As expected, there is a large discrepancy between the glass probe and the IrOx electrodes both calibrated with just the standard buffers. After using the spiked sputum data discussed above to adjust results, both sets of data match well with an average difference between the glass probe and IrOx electrodes of 0.067 pH. Further improvements can be made by fitting the calibration curve to a higher order polynomial than the simple linear fit used. These results also suggest that the sputum matrix is electrochemically similar between different patients since the calibration curve from a single sputum sample can recover the correct pH for all three samples without recalibrating for each patient. While the glass probe is still the go to pH electrode when absolute pH accuracy and consistency between different buffer types are desired, the IrOx electrodes are valuable in cases where the medium of the test solution does not change and miniature, inexpensive, and disposable sensors are favored over the bulky, fragile, and costly glass probes, such as for at-home sputum pH monitoring for CF patients.

Most importantly, these patient measurements also demonstrate that the entire smartphone-based pH sensing platform paired with either a standard glass probe or IrOx electrode performs as well as a commercial benchtop meter with a maximum variation between both sets of equipment of 0.031 pH. Hence, the smartphone-based pH sensor can achieve similar accuracy of a laboratory oriented tool, while at the same time being much more portable and amendable to point-of-care applications.

## 6. Conclusions

A POC-oriented, smartphone-based pH sensor was designed, built, and tested with both standard glass probes and IrOx electrodes for the application of monitoring pH levels of sputum in patients diagnosed with CF. The electronic peripheral module that attaches to the headphone port of the smartphone measures pH accurately when compared to a benchtop pH meter, all while being powered and controlled by the smartphone itself. Furthermore, the point-of-care oriented pH electrodes were shown to have a high sensitivity comparable to that of glass probes with acceptable amount stability given appropriate calibration. This complete, smartphone-based platform capable of dynamically interfacing with both types of sensors was shown to accurately measure actual sputum samples, which were verified with the benchtop meter. Hence, in terms of portability, accuracy, and flexibility, this platform has shown promise for practical at-home pH monitoring of lung health for CF patients. In the future, with this flexible platform as a launching pad, more CF biomarker sensors can be added to further improve the specificity and accuracy of this POC tool. 

## Supplementary Materials

The code for the smartphone application can be found online at https://github.com/acs009/smartphone-ph. The following are available online at http://www.mdpi.com/1424-8220/17/6/1245/s1, Figure S1: CAD images of 3D printed enclosure for electronic device, Figure S2: Sensor response to temperature variation and calibration of pH, Table S1: Bill of materials, Video S1: Smartphone pH Sensor Demo.
