# Supplementary material for: Smartphone-Based pH Sensor for Home Monitoring of Pulmonary Exacerbations in Cystic Fibrosis
Source: Sensors (Basel). 2017 May 30;17(6):1245. doi: 10.3390/s17061245 (PMC5491989; doi:10.3390/s17061245)
Supplement: Supplementary file 1 [file sensors-17-01245-s001.zip › sensors-192824 - Supplemental Materials.pdf]

Supplementary Data

Smartphone-Based pH Sensor for Home Monitoring of Pulmonary Exacerbations in Cystic Fibrosis

Alexander Sun [1], Tom Phelps [1], Chengyang Yao [1], A. G. Venkatesh [1], Douglas Conrad [2] and Drew A. Hall [1,*]

[1] Department of Electrical and Computer Engineering, University of California, San Diego, La Jolla, CA 92093, USA; acs009@ucsd.edu (A.S.); tphelps4432@gmail.com (T.P.); yaobryce@gmail.com (C.Y.); agvenkatesh@hotmail.com (A.G.V.)

[2] Department of Medicine, University of California, San Diego, La Jolla, CA 92093, USA; dconrad@ucsd.edu

* Correspondence: drewhall@ucsd.edu; Tel.: +1-858-534-3855

Contents

Supplementary Figures

Figure S1: CAD images of 3D printed enclosure for electronic device.
Figure S2: Sensor response to temperature variation and calibration of pH.

Supplementary Table

Table S1: Bill of materials

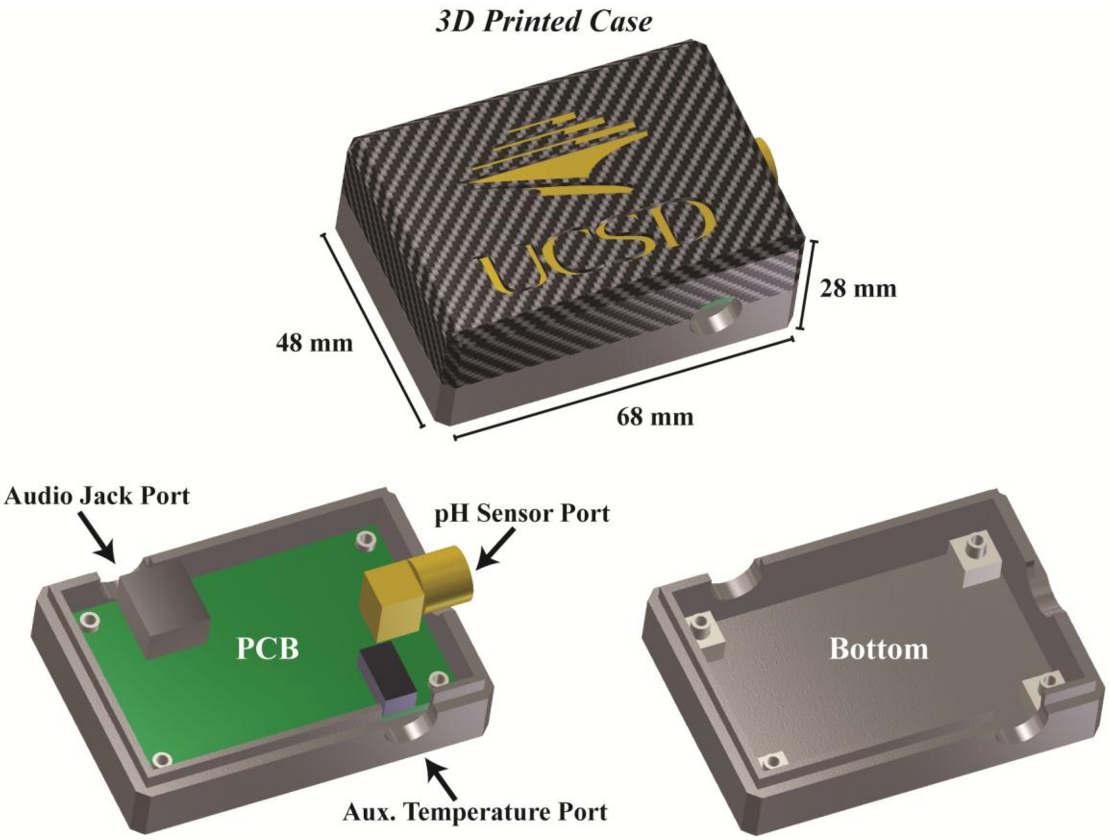

Figure S1. CAD images of 3D printed enclosure for the electronic device showing the complete device (top), uncovered with PCB (bottom left), and case bottom (bottom right).

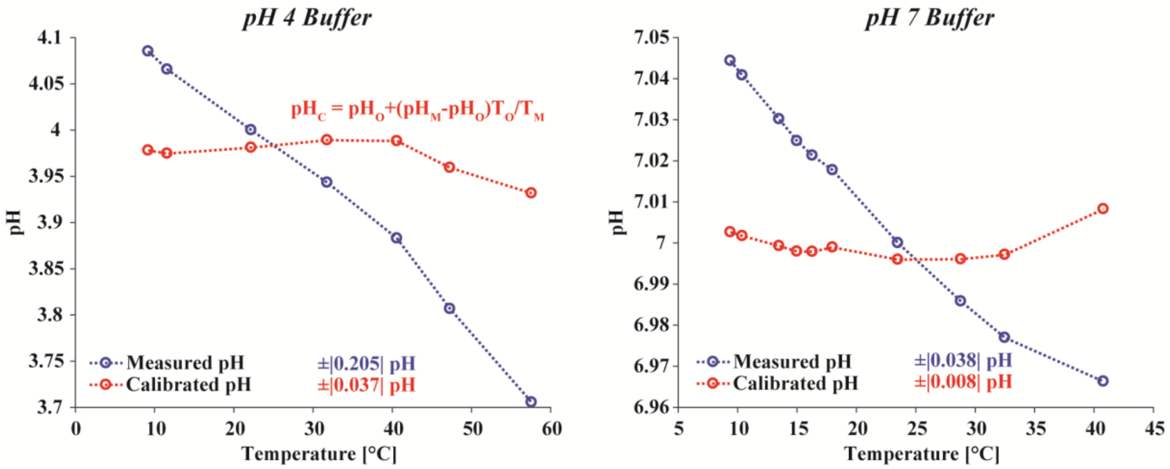

Figure S2. Sensor response to temperature variation and calibration of pH. This data was collected using the pH and temperature sensors submerged in both standard pH 4 and pH 7 buffers being heated and cooled externally. Without calibration, the maximum pH error reaches 0.205 and .038 pH for the pH 4 and 7 buffers, respectively. With calibration (pH_C), the variation is less than 0.037 and 0.008 pH, respectively. Since most sputum samples are approximately neutral and temperatures rarely deviate much from room temperature during testing, this error is minimal and temperature compensation is typically not required.

Table S1. Bill of materials

**Type**	**Company**	**Part #**	**Quantity**	**Price (@1000)**	**Total Price**
Comparator	NXP USA Inc.	NCX2200GW,125	2	0.15	$0.30
pH Sensor IC	Texas Instruments	LMP91200MT/NOPB	1	3.37	$3.37
OpAmp	Texas Instruments	OPA2333AMDREP	1	4.05	$4.05
Microcontroller	Microchip Technology	PIC16F690-I/ML	1	1.58	$1.58
Regulator IC	Linear Technology	LTC3632EMS8E#PBF	1	2.98	$2.98
Coupled Inductors	Coilcraft	LPR6235-123QMRC	1	1.21	$1.21
Schottky Diode	Diodes Incorporated	DFLS120L-7	2	0.175	$0.35
Switch	NXP USA Inc.	NX3L4053PW,118	1	0.383	$0.38
NMOS	Diodes Incorporated	ZXM61N03FTA	3	0.205	$0.62
PMOS	Diodes Incorporated	ZXM61P03FTA	3	0.205	$0.62
PCB Fabrication	-	-	1	1.566	$1.57
**Unit Cost** (@qty 1,000)					$17.02
